# Symbiotic cardiac pacemaker

**DOI:** 10.1038/s41467-019-09851-1

**Published:** 2019-04-23

**Authors:** Han Ouyang, Zhuo Liu, Ning Li, Bojing Shi, Yang Zou, Feng Xie, Ye Ma, Zhe Li, Hu Li, Qiang Zheng, Xuecheng Qu, Yubo Fan, Zhong Lin Wang, Hao Zhang, Zhou Li

**Affiliations:** 1 CAS Center for Excellence in Nanoscience, Beijing Key Laboratory of Micro-Nano Energy and Sensor, Beijing Institute of Nanoenergy and Nanosystems, Chinese Academy of Sciences, 100083 Beijing, China; 2 School of Nanoscience and Technology, University of Chinese Academy of Sciences, 100049 Beijing, China; 3 Beijing Advanced Innovation Center for Biomedical Engineering, School of Biological Science and Medical Engineering, Beihang University, 100083 Beijing, China; 4 Institute of Cardiothoracic Surgery at Changhai Hospital, Second Military Medical University, 200433 Shanghai, China; 5 School of Materials Science and Engineering, Georgia Institute of Technology, Atlanta, GA 30332-0245 USA

**Keywords:** Cardiac device therapy, Devices for energy harvesting, Biomedical engineering, Electronic devices

## Abstract

Self-powered implantable medical electronic devices that harvest biomechanical energy from cardiac motion, respiratory movement and blood flow are part of a paradigm shift that is on the horizon. Here, we demonstrate a fully implanted symbiotic pacemaker based on an implantable triboelectric nanogenerator, which achieves energy harvesting and storage as well as cardiac pacing on a large-animal scale. The symbiotic pacemaker successfully corrects sinus arrhythmia and prevents deterioration. The open circuit voltage of an implantable triboelectric nanogenerator reaches up to 65.2 V. The energy harvested from each cardiac motion cycle is 0.495 μJ, which is higher than the required endocardial pacing threshold energy (0.377 μJ). Implantable triboelectric nanogenerators for implantable medical devices offer advantages of excellent output performance, high power density, and good durability, and are expected to find application in fields of treatment and diagnosis as in vivo symbiotic bioelectronics.

## Abstract (web summary)

Implantable medical electronic devices are limited by battery lifetime and inflexibility, but self-powered devices can harvest biomechanical energy. Here the authors demonstrate cardiac pacing and correction of sinus arrhythmia with a symbiotic cardiac pacemaker, which is an implanted self-powered pacing system powered by cardiac motion, in a swine.

## Introduction

Millions of patients rely on implantable medical electronic devices (IMEDs) due to powerful diagnosis and treatment capabilities. For decades, in virtue of tremendous advances in micro/nano electronics technology, electronic circuits of IMEDs have evolved into ultra-low power consumption, miniaturized^1^, and flexible^2^ devices under the synergy between academia and industry^3–5^. However, batteries of IMEDs are generally bulky, rigid, and have short lifetimes owing to self-discharge, relatively low energy density, and inflexible packaging strategies^6^. Power source has impeded the progress of IMEDs^7,8^.

Various energy harvesters have been demonstrated in both providing complementary power to prolong the battery lifetime of IMEDs and providing independent power supplies^9–11^. These devices can harvest energy from heart beating^12–14^, muscle stretching^15–17^, glucose oxidation^18^, and endocochlear potential^19^ by exploiting piezo/triboelectric^20,21^, electromagnetic^13^, thermoelectric, and electrochemical effects. Among all of these routes, mechanical movement of organs is the most abundant energy source in vivo. Self-powered IMEDs harvesting biomechanical energy from cardiac motion^22–24^, respiratory movement, and blood flow is part of a paradigm shift that is on the horizon^25^. A triboelectric nanogenerator (TENG)^26^ has been developed as a potential biomechanical energy harvester for self-powered IMEDs^27^, which shows many unique advantages, such as flexibility, desirable biocompatibility, and light weight, in implantable applications^28^.

Implantable self-powered systems based on energy harvesters for physiological regulation have been applied to cardiac pacing^16,21,29^, deep brain stimulation^15^, nerves stimulation^30,31^, tissue engineering^32^ etc., in small-animal and cell scales (Supplementary Table 1). However, the energy required for small-animal and cell physiological regulation is much lower than that of humans. Meanwhile, the physiological regulation always needs controllable stimulating signals to guarantee the effects. For future clinical applications, it is urgent to develop high-output implantable energy harvesters for powering IMEDs with controllable output stimulating signals on human-scale animals for therapies^33,34^.

Inspired by the biological symbiosis phenomenon that involves interaction between different organisms living in close physical association, such as nitrogen-fixing bacteria with leguminous plants, we demonstrate an implanted symbiotic pacemaker (SPM) based on an implantable triboelectric nanogenerator (iTENG), which successfully achieves cardiac pacing and sinus arrhythmia correction on a large animal model. The iTENG-based cardiac pacemaker and the body form an interconnected symbiotic system. The SPM ingests energy from the body to maintain operation; meanwhile, the body obtains electrical stimulation from the SPM for the regulation of cardiac physiological activity. Both energy source and stimulus target of the SPM are the heart.

## Results

### System overview

**Fig. 1:**
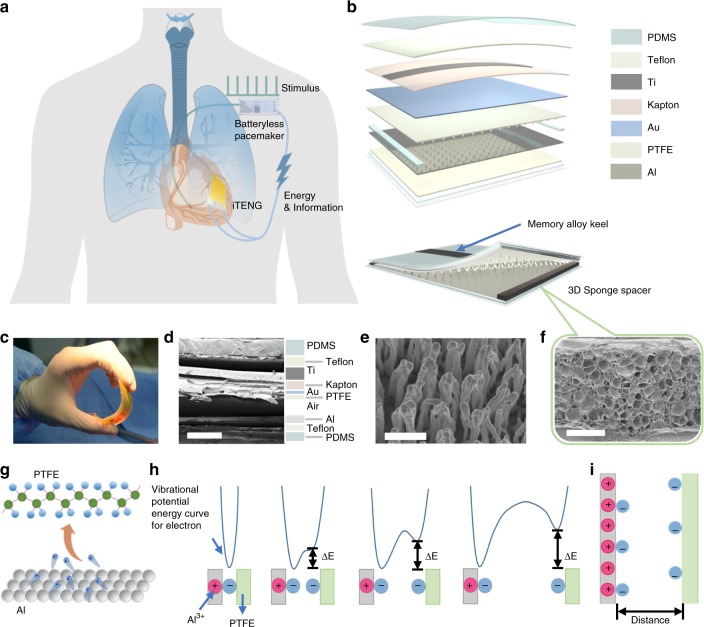
Overview of symbiotic pacemaker system. **a** Illustration of symbiotic cardiac pacemaker system. **b** Schematic structure diagram of implantable triboelectric nanogenerator (iTENG). **c** Photograph of iTENG under bending. **d** Cross-sectional scanning electron microscope (SEM) image of the iTENG (scale bar: 500 μm). **e** SEM images of the nanostructure on polytetrafluoroethylene (PTFE) film (scale bar: 1 μm). **f** SEM image of three dimensional (3D) elastic sponge structure (scale bar: 500 μm). **g**, **h** Schematic representation of the mechanism of charge transfer. **i** The model used to estimate the amount of charge separation that can arise from the transfer of charges

The SPM consisted of three parts: the energy harvest unit (iTENG), power management unit (PMU), and pacemaker unit. The energy harvest unit could harvest energy from cardiac motion. At first, switch of the PMU was turned off, the electricity generated by the energy harvest unit was stored in the capacitor of the PMU. Then, the switch was turned on by a magnet which was used as wireless passive trigger, the electrical energy could drive the pacemaker unit to produce pacing electrical pulses and control the rate of cardiac contraction (Fig. 1a). There was a core-shell structure of iTENG that consisted of two triboelectric layers, supporting structure and the shell with two encapsulation layers (Fig. 1b–d). Nanostructured polytetrafluoroethylene (PTFE) thin film was employed as one triboelectric layer (Fig. 1e). A three dimensional (3D) elastic sponge (ethylene-vinyl acetate copolymer, EVA) played a role as a spacer (Fig. 1f), and a memory alloy ribbon (highly resilient titanium) was utilized as the keel. The iTENG was entirely packaged by a flexible Teflon film and a polydimethylsiloxane (PDMS) layer to enhance its structural stability and avoid environmental liquid damage to the device.

The operating principle of the iTENG was based on the coupling of contact electrification and electrostatic induction. Contact electrification is mainly caused by the transfer of surface electrons^35^ (Fig. 1g–i, Supplementary Note 1). Then an electrical potential between two triboelectric layers drove the electrons through external loads due to electrostatic induction.

### Characterization of the implantable triboelectric nanogenerator

**Fig. 2:**
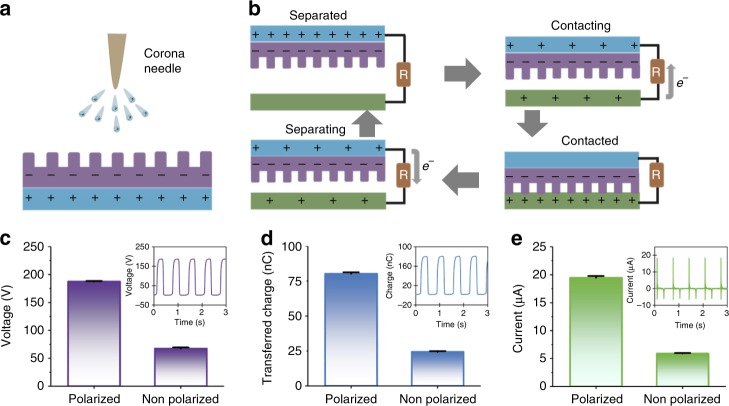
Polarized polytetrafluoroethylene film based triboelectric nanogenerator. **a** Sketch of a corona discharge system. **b** Schematic diagram of the working principle of iTENG. **c**–**e** The output voltage, transferred charge and current of polarized and non-polarized PTFE film based TENG driven by a linear motor. Source data of **c**–**e** are provided as a Source Data file. All data in **c**–**e** are presented as mean ± s.d.

The effective contact area and surface charge density have significant impacts on the TENG output^26,36^. The nanostructure of PTFE and spacer/keel supporting structure could effectively increase the contact area to improve output performance of the TENG^37^ (Supplementary Note 2, Supplementary Table 2, Supplementary Fig. 1). Corona discharge method was used to increase the surface charge density of the PTFE triboelectric layer, which can enhance the output of TENG as well (Fig. 2a). The current flowed from corona needle with high potential into the air, by ionizing and creating a region of plasma around the needle. The ions eventually passed charge to areas of lower potential (PTFE film). A mechanical linear motor was employed to characterize the effect of corona polarization on the electrical output of the TENG. The *V*_*OC*_ (open-circuit voltage), *Q*_*SC*_ (short-circuit transferred charge), and the corresponding *I*_*SC*_ (short-circuit current) of group polarized were up to 187 V, 80.2 nC, and 19.5 μA, respectively. In comparison, the non-polarized ones were 67.5 V, 24.8 nC, and 5.9 μA accordingly. The TENGs with unified specification were utilized in this experiment (Fig. 2b–e).

**Fig. 3:**
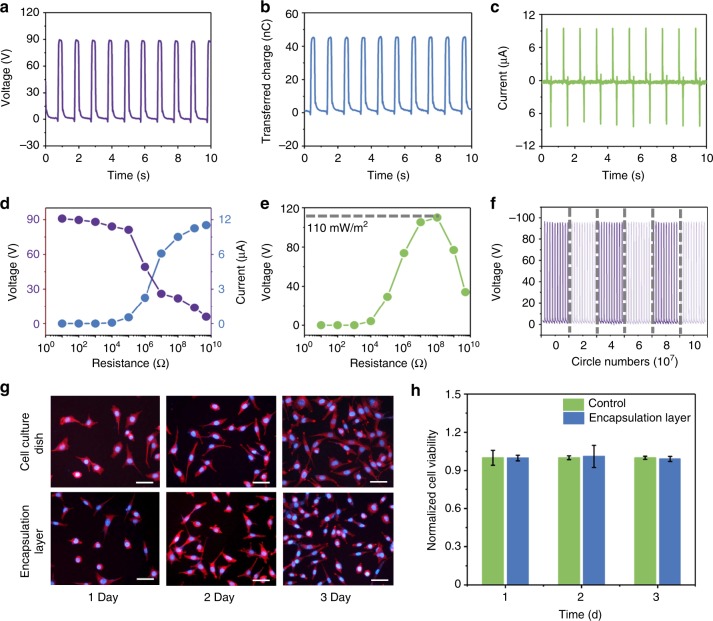
In vitro evaluation of the implantable triboelectric nanogenerator. **a**–**c** Open-circuit voltage, transferred charge and short-circuit current of the iTENG driven by a linear motor. **d** Voltage and current at different load resistances. **e** Peak power density at different load resistances. **f** Stability tests of iTENG. **g** Fluorescence images of stained L929 cells that were cultured on encapsulation layers of the TENG; the scale bar is 50 μm. **h** The normalized viability of L929 cells after being cultured for 3 days. Source data of **h** are provided as a Source Data file. All data in **h** are presented as mean ± s.d.

Then the polarized PTFE-based TENG was hermetically sealed by flexible encapsulation layers to fabricate an iTENG. The linear motor was used to simulate low-frequency biomechanical excitation for testing the in vitro electrical output performance of the iTENG. The average values of *V*_*OC*_, *Q*_*SC*_ and the *I*_*SC*_ were 97.5 V, 49.1 nC, and 10.1 μA, respectively (Fig. 3a–c). Further investigations of the effective electric power of the iTENG showed that the instantaneous current decreased and voltage rose with increase of the load resistances (Fig. 3d). Hence, a peak power density of 110 mW m^−2^ was achieved at a load resistance of 100 MΩ (Fig. 3e).

Superior electrical output, biocompatibility, and stability are critical aspects for an in vivo energy harvester. An accelerate fatigue test was employed to evaluate the long-term output performance of the iTENG. After 100 million mechanical stimuli cycles by vibration table, *V*_*OC*_ of the iTENG driven by linear motor was maintained stably at 95 V compared with its initial state, exhibiting outstanding durability and stability (Fig. 3f). In addition, to explore the impact of the ionic liquid environment (mimicking the in vivo environment) on iTENG long-term operation, the test environment was replaced with phosphate buffer saline (PBS 1×). After accelerate fatigue test in PBS for 100 million cycles, the output voltage, transferred charge and current of the iTENG were about 93 V, 47 nC, 9 μA, respectively, when tested in dry environment (relative humidity 40–50%) and 91 V, 45 nC, 8 μA when tested in PBS solution (Supplementary Fig. 2a–f). There is no water penetration and damage on the iTENG (Supplementary Fig. 3a–e). The encapsulation layer can effectively avoid negative effects of wet conditions on output of the iTENG.

Excellent biocompatibility is essential for IMEDs to avoid an adverse influence on the surrounding tissue. As an overview for the biocompatibility and cytotoxicity of iTENG, we observed growth, and viability of Mouse fibroblast (L929s) on the above encapsulation layer material and the cell culture dish. L929s adhered to both of the materials, with similar spreading, and intact detectable cellular structures, i.e. cell nucleus and actin microfilaments (Fig. 3g). The MTT (3-(4,5-dimethylthiazol-2-yl)-2,5-diphenyl-2H-tetrazolium bromide) value of the experiment group was similar to the value of the control group after 3 days of culture (Fig. 3h). These results demonstrated the good cytocompatibility of the iTENG.

### I**n vivo** performance of the implantable triboelectric nanogenerator

**Fig. 4:**
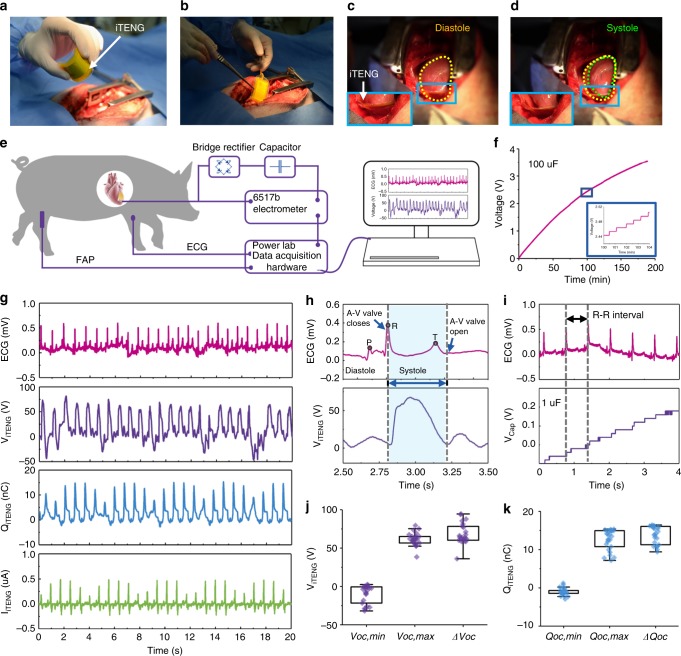
In vivo energy harvest and electrical characterization. **a**, **b** The iTENG implantation process in animal experiments. **c**, **d** The iTENG was driven by the diastole and systole of the heart. **e** Schematic of in vivo experimental electrical characterizations. **f** Charging curve of a 100 μF capacitor charged by iTENG. **g** In vivo open-circuit voltage, transferred charge, short-circuit current of the iTENG and simultaneously recorded electrocardiography (ECG). **h** In vivo output open-circuit voltage and simultaneously recorded ECG signals. **i** The relationship between ECG signals and the voltage of a 1 μF capacitor charged by iTENG. **j** Statistics-analysis of minimum voltage, maximum voltage, and the voltage difference. **k** Statistics-analysis of minimum transferred charge, maximum transferred charge, and the transferred charge difference. Source data of **j**, **k** are provided as a Source Data file. All data in **j**, **k** are presented as mean ± s.d.

To evaluate the performance of the iTENG as the energy harvest unit in vivo, a large animal model (Adult Yorkshire porcine, male, 45 kg) was employed in this work (Fig. 4a–e). The iTENG was placed between the heart and pericardium, and the PTFE side faced the left ventricular wall. Cardiac motion caused periodic contact and separation of the two triboelectric layers. The electrical energy generated by iTENG was stored in a 100 μF capacitor through a rectifier. As a result, the voltage of capacitor could be charged from 0 to 3.55 V within 190 min under conditions of blood pressure of ~100/70 mmHg and heart rate of ~77 bpm (Fig. 4f, Supplementary Fig. 4, Supplementary Movie 1). Here, in vivo *V*_*OC*_ was up to ~65.2 V, *Q*_*SC*_ was ~13.6 nC, and the corresponding *I*_*SC*_ was ~0.5 μA (Fig. 4g, Supplementary Fig. 5a–c).

In addition, the electrical output of iTENG was completely synchronized with the corresponding electrocardiography (ECG) (Fig. 4h). The rise inflection point of the voltage of the iTENG was consistent with the peak of R wave. When the atrioventricular (A–V) valves closed, the heart turned into phase of systole. The iTENG was compressed and produced a voltage pulse with the width equivalent to phase of systole (Supplementary Fig. 6a, b). Furthermore, the rise inflection point of the current of the iTENG was consistent with the peak of R wave, and the peak of the current signal was synchronized with S wave (Supplementary Fig. 6d, e, Supplementary Note. 3). The voltage of the capacitor was simultaneously stepwise increasing with each cardiac cycle (Fig. 4i, Supplementary Fig. 7). The generated energy during each cycle was *E*_*max*=_0.495 μJ. Here, the average values of *V*_*OC,max*_, *V*_*OC,min*_, and *ΔV*_*OC*_ were 65.2 V, −7.7 V, and 72.9 V, respectively. The average values of *Q*_*SC,max*_, *Q*_*SC,min*_ and *ΔQ*_*SC*_ were 13.6 nC, −1 nC, and 14.6 nC, respectively (Fig. 4j, k, Supplementary Note 4).

To present an intuitive view of the iTENG powering electronic devices, the energy harvested by the iTENG from cardiac motion was stored in a capacitor (100 μF, 3.55 V) to drive a commercial pacemaker. The commercial pacemaker generated a series of pacing electrical pulses with a voltage of about 4 V and a pulse width of 0.9 ms (Supplementary Fig. 8a–d, Supplementary Note 8). A light-emitting diode (LED) was also directly connected to the implanted iTENG. The LED blinked synchronously with heart beating (Supplementary Fig. 9a–c, Supplementary Movie 2, Supplementary Note 9).

### Pacing in large animal model by symbiotic pacemaker in vivo

**Fig. 5:**
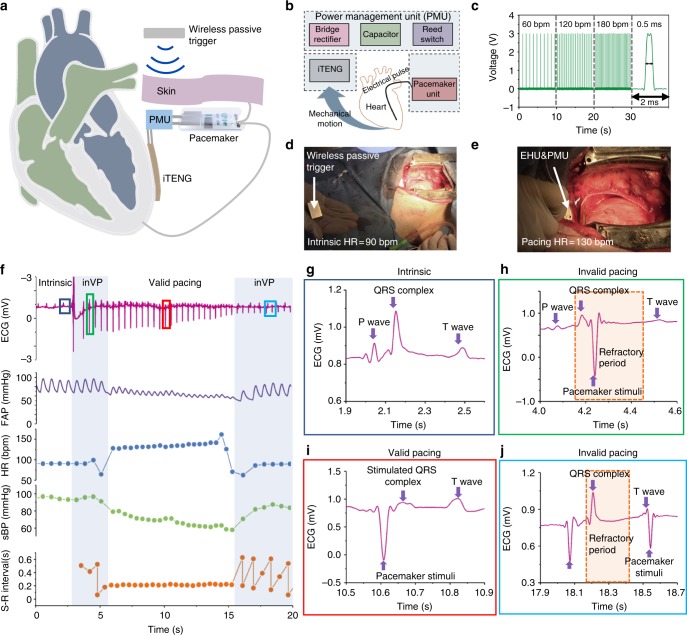
symbiotic cardiac pacing in vivo. **a** Illustration of symbiotic cardiac pacemaker system turned on by wireless passive trigger. **b** A block diagram of the components in symbiotic cardiac pacemaker system. **c** Stimulation pulse with different frequencies generated by pacemaker unit. **d**, **e** Symbiotic cardiac pacemaker system turned on by wireless passive trigger in animal experiments. **f** ECG, Femoral Artery Pressure (FAP) and heart rate (HR) and systolic blood pressure (sBP), Stimulus-R wave (S-R) interval during symbiotic cardiac pacemaker system work. **g** ECG of the intrinsic heart rate, with a normal sBP. **h** ECG with a pacing stimulus in the refractory period, with a normal sBP. **i** ECG of successful pacing, with a significantly decreased sBP. **j** ECG with failed pacing by attenuated stimuli, with a restored sBP

The iTENG was connected to the PMU via wires and rectifier. The electrical energy generated from the iTENG was stored in the capacitor of the PMU, which started to power the pacemaker unit after the magnet placed outside the body turned on the reed switch (Fig. 5a, b, Supplementary Fig. 10). The generated electrical pulses from the pacemaker unit can induce myocardial contraction and regulate heart rate through pacing electrodes. The output voltage and duration of the electrical pulses were 3 V and 0.5 ms respectively (Fig. 5c). The rate of the electrical pulses was preset to 130 bpm, in consideration of the high heart rate of the pig during the experiment.

To achieve cardiac pacing in a large animal model, we implanted the entire SPM system into the chest of a pig. After continuously harvesting energy from cardiac motion in ~200 min, the SPM was switched on by the wireless passive trigger (NdFeB magnet). The electrical pulses (0.5 ms, 130 bpm; setting parameter) were generated and transmitted to the myocardium (Fig. 5d, e). Some typical exhibitions of ECG had appeared during the pacing process by SPM (Fig. 5f). Here, cardiac physiology states of experimental animals can be divided into four periods, including intrinsic rhythm, invalid pacing, valid pacing and invalid pacing period (Supplementary Movie 3).

The typical P wave, QRS complex and T wave could be identified in the ECG of intrinsic rhythm (Fig. 5g). When the SPM was switched on and releasing the pacing stimuli in the refractory period, the heart had no response to the stimuli and showed an ECG of invalid pacing (Fig. 5h). Once the pacing stimuli released in the non-refractory period, a contraction of heart and a stimulated QRS complex could be observed in the ECG. Here, the stimulated QRS complex appeared immediately following the pacing stimulus, indicating the heart was successfully paced by SPM (Fig. 5i). With the consumption of the energy by fast pacing, the voltage of the capacitor of the PMU and the amplitude of the pacing electrical pulse declined concomitantly, leading to failure in pacing the heart. The pacing stimulus could not induce a stimulated QRS complex in ECG and an invalid pacing ECG could be detected (Fig. 5j).

The variations of heart rate and blood pressure of the experimental animal further illustrated the above-described process. The heart rate of experimental animal was increased from 90 bpm in the intrinsic phase to ~130 bpm in the valid pacing phase and then returned to 90 bpm during the invalid pacing phase. The systolic blood pressure declined significantly from ~100 mmHg of the intrinsic phase to ~60 mmHg of the valid pacing phase due to the fast pacing by the SPM. It returned to ~90 mmHg when the heart rate slowed down in the invalid phase. The above results confirmed that the SPM system successfully achieved cardiac pacing in large animal scale.

### Correcting arrhythmia in large animal model

**Fig. 6:**
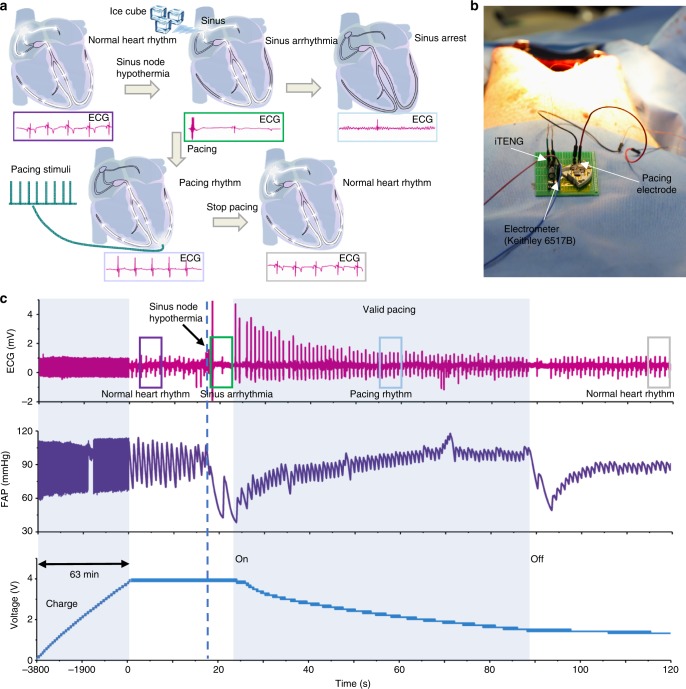
Correcting arrhythmia on large animal model. **a** Illustration of symbiotic cardiac pacemaker system correcting arrhythmia on large animal model. **b** Symbiotic cardiac pacemaker system in animal experiments. **c** electrocardiography (ECG), Femoral Artery Pressure (FAP) of the animal model and voltage of capacitor during correcting arrhythmia experiment

To demonstrate the ability of SPM to correct arrhythmia, we performed pacing therapy on an animal model (Adult Yorkshire porcine, male, 35 kg) with sinus arrhythmia induced by sinus node hypothermia^38,39^. The arrhythmia induced by sinus node hypothermia may deteriorate to sinus arrest and even ventricular fibrillation, which would cause death if not treated promptly (Fig. 6a, Supplementary Fig. 11a–c).

The electrical energy generated by the iTENG was stored in a 200 μF capacitor for powering the pacemaker. As a result, the voltage of the capacitor can be charged from 0 to ~4 V within 63 min under the blood pressure of ~110/60 mmHg and heart rate of ~82 bpm (Fig. 6b, c). Here, in vivo *V*_*OC*_ was up to ~39 V, *Q*_*SC*_ was ~21 nC, and the corresponding *I*_*SC*_ was ~0.8 μA (Supplementary Fig. 12a–c). In addition, the output voltage and power have been evaluated under resting (∼50 bpm), active (∼90 bpm), and stressing (∼130 bpm) states (Supplementary Fig. 13, Supplementary Note 6).

Ice cube was used to create sinus node hypothermia, then a typical arrhythmia ECG was observed. The pacing therapy was performed promptly. Sinus arrhythmia was converted to pacing rhythm when the iTENG-based SPM was turned on. Heart rate remained at ~68 bpm, and blood pressure began to recover to the previous level. After about one minute, the power supply voltage dropped to 1.4 V. The SPM stopped working, and the pacing rhythm turned into normal heart rhythm. This result confirmed that SPM successfully corrected sinus arrhythmia and prevented further deteriorating condition (Fig. 6c).

## Discussion

In summary, we demonstrate a symbiotic pacemaker (SPM) and successfully achieve cardiac pacing in a large animal model. The ability of the SPM to correct sinus arrhythmia and prevent the deteriorating condition has also been proven. Furthermore, the in vivo output performance of the present iTENG is impressive, as it is more than four times as large as the previous output records^27^ (Supplementary Table 3). The energy harvested from each cardiac cycle is 0.495 μJ, which is higher than the pacing threshold energy of pigs^40^ (0.262 μJ) and humans^41^ (0.377 μJ, Medtronic’s Micra TPS on human, Supplementary Note 5).

The iTENG provides a promising method to harvest in vivo biomechanical energy, with advantages of wide choice of materials, high outputs, good flexibility, light weight, excellent durability and low cost (Supplementary Table 4, Supplementary Fig. 14). In addition, the iTENG has shown remarkable mechanical durability (100 million mechanical stimuli cycles) and cytocompatibility, which are determinants for long-term implantable devices. Furthermore, the iTENG is adapted for harvesting low-frequency vibrational energy^42^, especially in vivo.

Some challenges still need to be overcome for the iTENG and SPM to reach clinical applications. To meet requirements for a minimally invasive implantation process and for better comfort with long-term in vivo operation, it is necessary to develop an iTENG with characteristics of small size, high energy density, efficient fixation with bio-tissue and long-term biosafety. Such developments depend on materials science, micro/nano-fabrication technologies and electronic techniques. In addition, a more efficient power management unit adapted to an iTENG is significant for further development of SPM^43^.

In addition to cardiac pacing, for other in vivo applications of phototherapy and cardiovascular events identifications, an iTENG may act as a direct electrical stimulation source for tissue engineering^32^, nerve regeneration^28^, and stem cell differentiation^44^ since the output voltage of the iTENG is higher than that of cells and tissues^45^. Meanwhile, the low current of the iTENG can avoid adverse effects on cells or tissues.

Symbiotic bioelectronics and the body form an interconnected symbiotic system. Both the energy source and stimulus target of the symbiotic device is the body. The symbiotic bioelectronics expects to be applied to treatment and rehabilitation, such as cardiac pacing, nerve stimulation, tissue repair, and even cell differentiation, in a self-powered approach.

## Methods

### Fabrication of triboelectric layer for implantable triboelectric nanogenerator

The fabricated nanostructured PTFE film was processed by inductively coupled plasma etching system (SENTECH/SI 500). A piece of 50 μm PTFE film was rinsed with alcohol and deionized water. The Au (Aurum), which acted as the mask for the etching process, was sputtered onto the PTFE surface for about 30 s. Then, this PTFE film was etched by ICP reactive ion etching for 300 s (ICP power: 400 W and 100 W, respectively). The reaction gas in the ICP process was CF_4_ (30.0 sccm), O_2_ (10.0 sccm), and Ar (15.0 sccm). Finally, the Au electrode (50 nm) was deposited by magnetron sputter (Denton Discovery 635) for 15 min (sputter power 100 W). Au bottom electrode connected by wire to ground and a polarization voltage of 5 kV was applied for 15 min through the corona needle.

### Encapsulation of the implantable triboelectric nanogenerator

The teflon film was employed as a first package layer. Then PDMS (Dow Corning Sylgard 184 Silicone Encapsulant) was mixed with the curing agent (10:1), which was spin-coated on Teflon as the second package layer, and then solidified at 80 °C for an hour. The spacing of the two triboelectric layers was kept at about 500 μm after pre-compression and encapsulation.

### Characterization methods

All scanning electron microscopy (SEM) images were taken with a Hitachi field emission scanning electron microscope (SU 8020). The voltage, transferred charge and current were detected by an electrometer (Keithley 6517B) and recorded by oscilloscope (Teledyne LeCroy HD 4096) or data acquisition hardware (PowerLab 4/35).

### Calculation of a peak power density

Peak power density (PPD) is employed to evaluate the generator output performance. The iTENG was connected with different loads, then the voltages of the loads and the currents in the circuit were tested separately. The PPD can be derived by the following equation.

1 $${\mathrm{PPD}} = \frac{{V_{\mathrm{peak}} \times I_{\mathrm{peak}}}}{S}$$

Where, *V*_peak_ is the peak value of the voltage of the load and *I*_peak_ is the peak value of the corresponding current in the circuit. *S* represents the area of triboelectric layer.

### In vitro test

The iTENG was driven by a linear motor (frequency, 1 Hz, operating distance, 50 mm; acceleration, 1 m/s^2^; deceleration, 1 m/s^2^; maximum speed,1 m/s^2^). The applied strain *ε* can be derived by the following equation.

2 $$\varepsilon = \frac{h}{{2R}}$$

Where *h* was the thickness of iTENG and *R* was the bending radius. Thus, the applied strain on the iTENG was 0.16 %. The applied force was about 40 N, measured by a dynamometer (MARK-10-M7-2)

### Accelerated fatigue test in vitro

Vibration table (VT-500, YMC PIEZORONICS.INC) was employed as a source of mechanical stimuli for saturated accelerated fatigue test (operating distance, 1.5 mm; frequency, 100 Hz). The output voltage was measured every 10^7^ cycles in atmosphere (relative humidity 40–50%). The measuring method was shown in the part of “In vitro test.”

### Accelerated fatigue test in liquid

The accelerated fatigue test in liquid is for stimulating the in vivo environment. The iTENG (39 × 61 × 0.99 mm) was placed at the bottom of the sealed chamber (55 × 80 × 23 mm) filled with the phosphate buffer saline (PBS 1×), and then a mass loading (45 g, 32 × 53 × 13 mm, rubber) was put on the iTENG. The test process was similar to part of “accelerated fatigue test”. The output for iTENG was measured in an atomospheric environment (relative humidity 40–50%) and PBS solution.

### Cell culture

The L929 cells (Central South University, Hunan, China) were cultured with RPMI medium 1640 basic (1×), 10% fetal bovine serum (Gibco) and supplemented with 1% penicillin-streptomycin solution (Life Technologies, Shanghai, China) in a 75 cm^2^ flask. The culture conditions remained in a humidified atmosphere with 5% CO_2_ at 37 °C. L929 cells were seeded in 24-well plates after 3 days. The cells of the experimental group were exposed to the encapsulation layer film on the 24-well plates. MTT assay was employed to evaluate the proliferation of cultured L929 cells. The analytical assays were performed at day 1, day 2, and day 3. At least three wells were randomly examined each time.

### Cell morphology and immunofluorescent staining

Phalloidin and DAPI were employed to stain the cytoskeleton and nucleus respectively. Immunohistochemically fixed fluid (Beyotime) be used to fix the samples for 30 min. Next, the samples were rinsed three times with prewarmed PBS (1×). The 0.1 % bovine serum albumin solution was employed to block the samples for 1 h at 37 °C and the samples were incubated for 2 h at 37 °C with Alexa Fluor phalloidin 568 conjugate (1:200 dilution) and DAPI (1:400 dilution). Inversion fluorescence microscope was used to obvious samples.

### Normalized cell viability

The data reducing method of normalized is as follow:

3 $$\overline {M_{\mathrm{C}}} = \frac{{\mathop {\sum }\nolimits_1^n M_{{\mathrm{Cn}}}}}{n}$$

4 $$\overline {M_{\mathrm{E}}} = \frac{{\mathop {\sum }\nolimits_1^n M_{{\mathrm{En}}}}}{n}$$

5 $${\mathrm{NCV}_{{\mathrm{C}}}} = \frac{{\overline {M}_{{\mathrm{C}}} }}{{\overline {M}_{{\mathrm{C}}} }} \times 100{\mathrm{\% }}$$

6 $${\mathrm{NCV}_{{\mathrm{E}}}} = \frac{{\overline {M}_{{\mathrm{E}}} }}{{\overline {M}_{{\mathrm{C}}} }} \times 100{\mathrm{\% }}$$

Here, $\overline {M_{\mathrm{c}}}$ is the MTT mean value of control group,$\overline {M_{\mathrm{E}}}$ is the MTT mean value of experiment group. NCV_C_ represents the normalized cell viability of control group, NCV_E_ stands for the normalized cell viability of Experiment group.

### Large animal model experiment

The experimental process was strictly in line with the “Shanghai Administration Rule of Laboratory Animal” and the Institutional Animal Care and Use Committee (IACUC) approved protocol of the Animal Care Center at the Second Military Medical University.

### In vivo energy harvesting

The male Yorkshire porcine (45 kg) fasted for 12 h before surgery. Briefly, the animal was anesthetized with an injection of ketamine (8 mg/kg, IM), followed by propofol (1 mg/kg, IV), and then intratracheally intubated and ventilated. Anesthesia was maintained with 4 mg kg^−1^ h^−1^ propofol during the surgery. A dynamic arterial pressure catheter was inserted into the right femoral artery and linked the data acquisition hardware (PowerLab 4/35) for femoral artery pressure (FAP) measurement. The ECG was recorded by data acquisition hardware. Next, iTENG was implanted between the heart and pericardium with its PTFE side facing the left ventricular front wall. The iTENG was then connected to a capacitor through a rectifier and/or connected to the electrometer (Keithley 6517B) to measure the electric output and recorded by data acquisition hardware (PowerLab 4/35).

### Pacing experiments

The symbiotic pacemaker system consisted of three parts: the energy harvest unit, power management unit and pacemaker unit. The pacemaker unit was implanted subcutaneous with a depth of 2–3 cm and produced the stimulus pulses signals. The iTENG was connected to the power management unit, which consists of a rectifier (DB107, SEP Electronic Corp.), a capacitor (100 μF, Risym), and a reed switch (GPS-14A, BASEUS). The electricity generated by the iTENG was stored in the capacitor through a rectifier. When switched on controlled by the wireless passive trigger, electric energy powered the pacemaker unit to produce the pacing stimuli.

### Wireless passive trigger process

The electricity generated by the iTENG was stored in the capacitor of PMU through a rectifier and wires. The capacitor was directly connected to the pacemaker unit via wires and reed switch that was controlled by the static magnetic field induced by a NdFeB magnet. When the magnet was close to the reed switch, the switch was turned on and the capacitor powered the pacemaker unit. When the magnet was away from the reed switch, it was turned off. Here, the pacemaker unit and power management unit were implanted 2–3 cm under the skin.

### Correcting arrhythmia experiments

The male Yorkshire porcine (35 kg) fasted for 12 h before surgery. Briefly, the animal was anesthetized with an injection of ketamine (8 mg/kg, IM), followed by propofol (1 mg/kg, IV), and then intratracheally intubated and ventilated. The iTENG was placed in the space between the heart and the pericardium. The stitches were penetrated through the pericardium to form criss-crossing near the four corners of the iTENG to make it be fixed on the pericardium. Then the pericardium was sutured closed at the end of implantation.

Anesthesia was maintained with 4 mg kg^−1^ h^−1^ propofol during the surgery. The ice cube (about 3–5 g) was placed near the sinus node to induce bradycardia arrhythmia. The iTENG was connected to the power management unit, which consisted of a rectifier (DB107, SEP Electronic Corp.) and a capacitor (200 μF, Risym). The electricity generated by the iTENG was stored in the capacitor through a rectifier. The voltage value of the capacitor was measured by the electrometer (Keithley 6517B) and recorded by data acquisition hardware (PowerLab 4/35). The electric energy stored in the capacitor can power the pacemaker unit to produce the pacing stimuli.

## Supplementary information

Supplementary Information

Source Data

Supplementary Movie 1

Supplementary Movie 2

Supplementary Movie 3

Reporting Summary

Description of Additional Supplementary Files

## Data Availability

All data needed to evaluate the conclusions in the paper are present in the paper and/or the Supplementary Information. The source data underlying Figs. 2c–e, 3h, and 4j–k and Supplementary Figs. 5c–f are provided as a Source Data file (10.6084/m9.figshare.7763453). Additional data related to this paper may be requested from the authors.
