# Supplementary material for: Symbiotic cardiac pacemaker
Source: Nat Commun. 2019 Apr 23;10:1821. doi: 10.1038/s41467-019-09851-1 (PMC6478903; doi:10.1038/s41467-019-09851-1)
Supplement: Supplementary file 1 — Supplementary Information [file 41467_2019_9851_MOESM1_ESM.pdf]

Supplementary Information

Symbiotic Cardiac Pacemaker

Ouyang et al.

Supplementary Figures

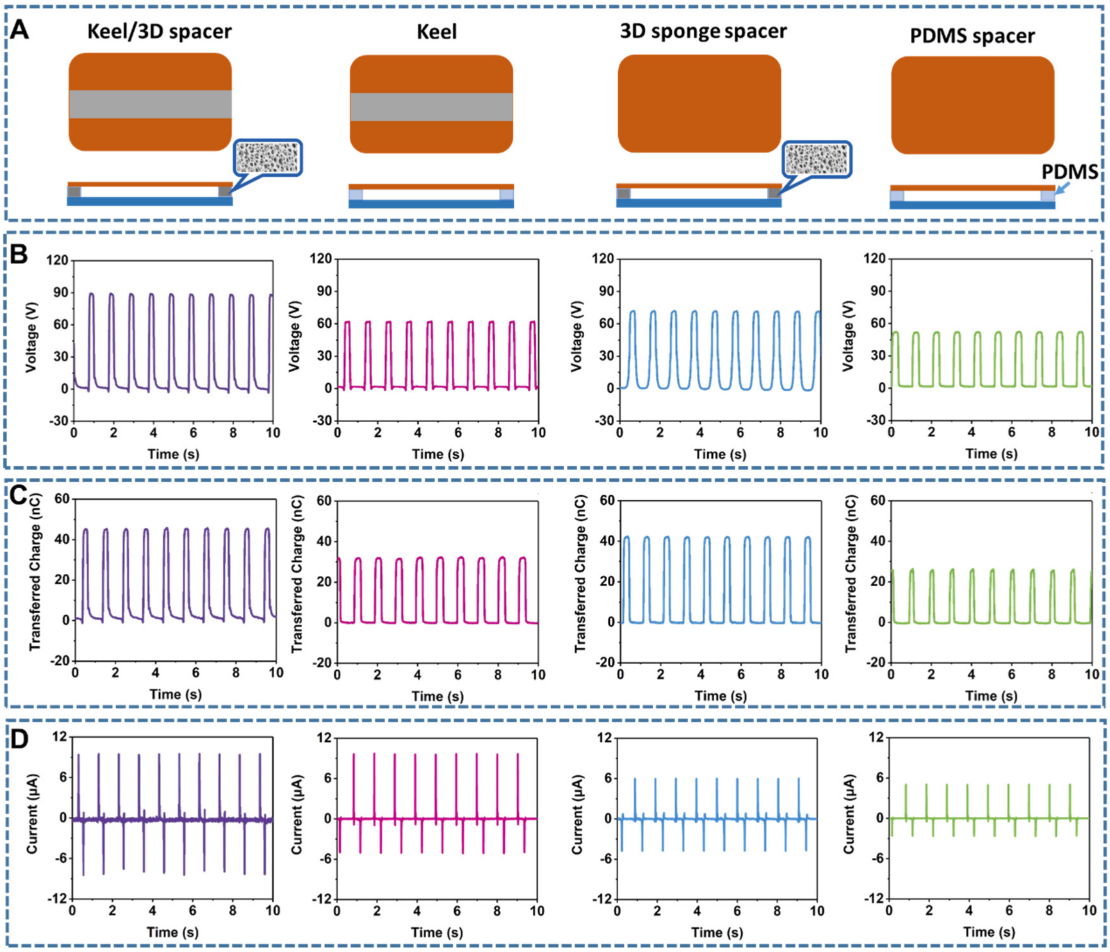

Supplementary Figure 1 Electrical output of iTENG with different supporting structures. (a) Schematic of iTENG with different supporting structures (memory alloy keel/3D sponge spacer, memory alloy keel, 3D sponge spacer and PDMS spacer supporting structure). Open-circuit voltage (b), transferred charge (c) and short-circuit current (d) of iTENG with different supporting structures driven by a linear motor.

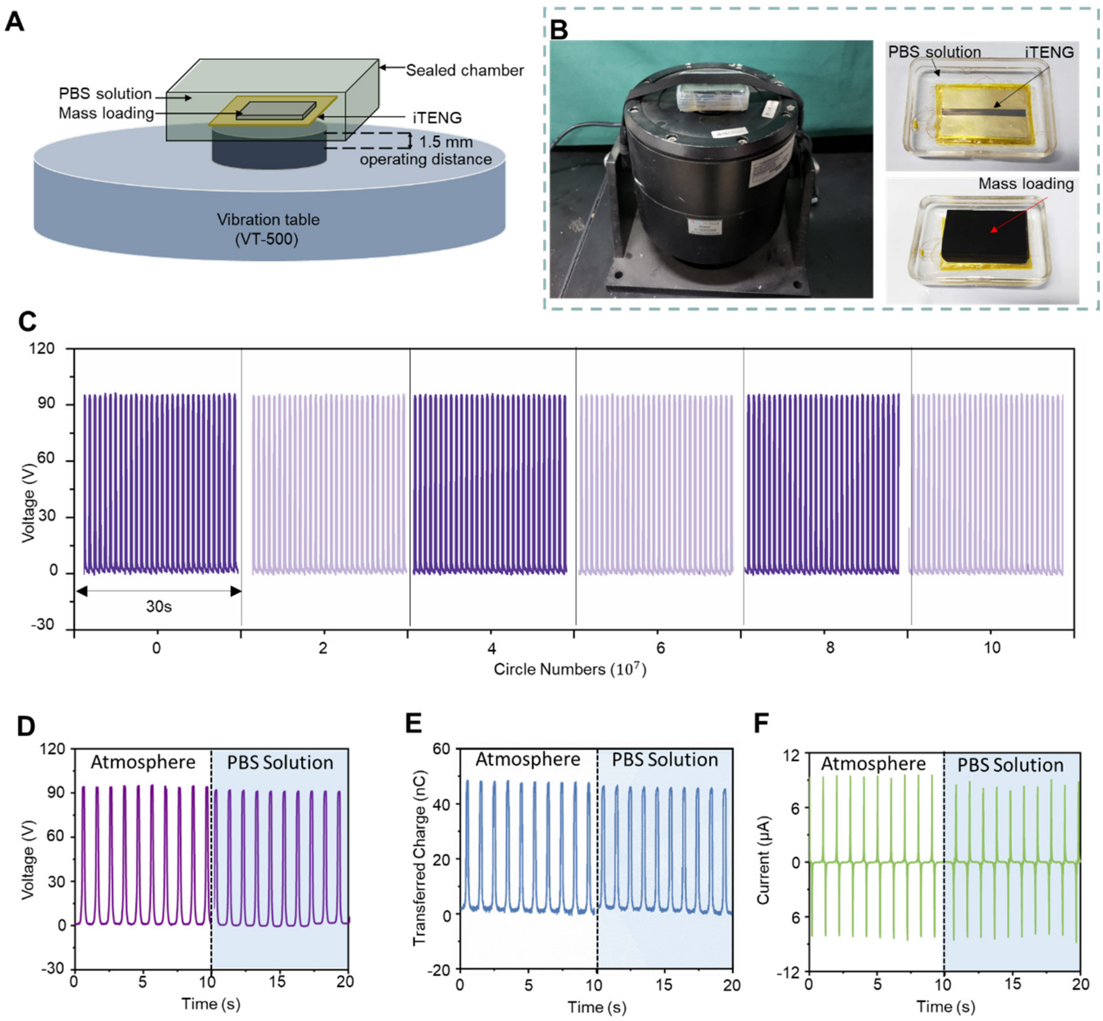

Supplementary Figure 2 Illustration (a) and photographs (b) of accelerated fatigue test. (c) Open-circuit voltage of the iTENG and its durability test. Open-circuit voltage (d), short-circuit transferred charge (e) and short-circuit current (f) of iTENG in atmosphere and liquid environment (PBS 1×).

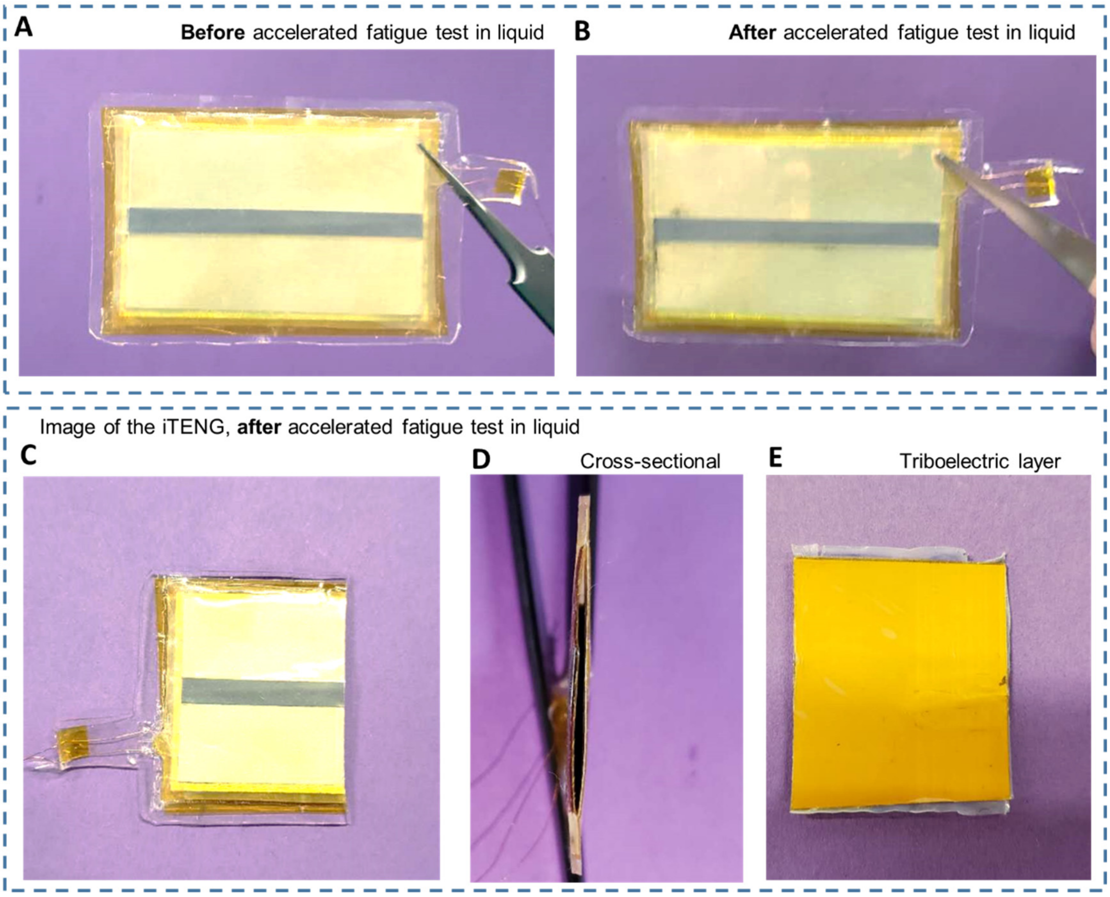

Supplementary Figure 3 Photographs of iTENG. Before (a) and after (b) accelerated fatigue test in liquid. The image of dismantled iTENG (c, d, e).

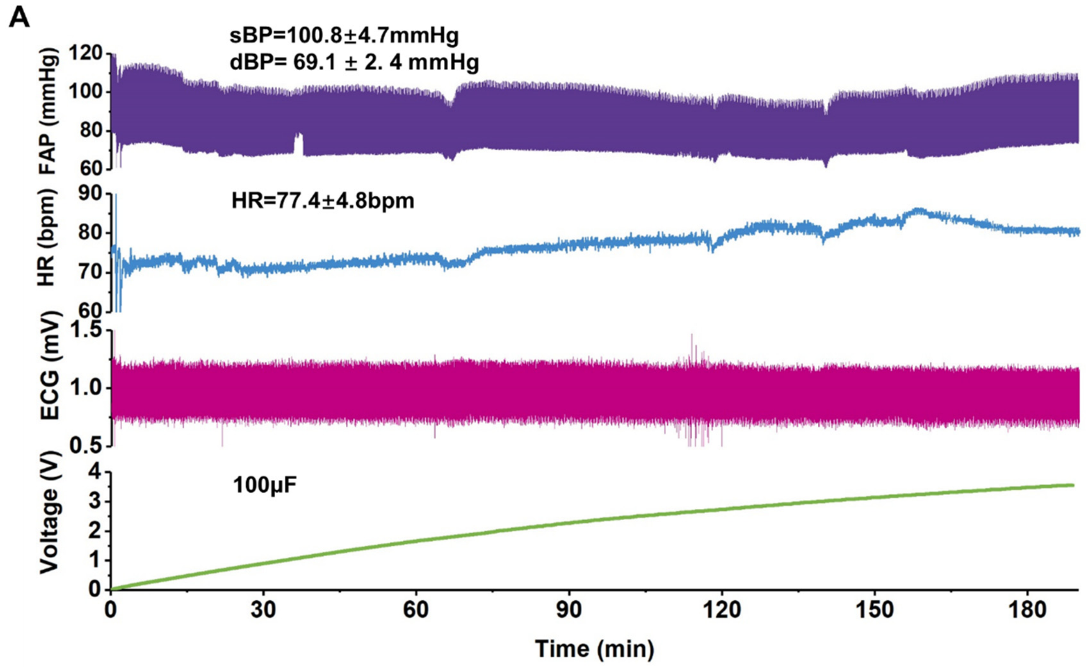

Supplementary Figure 4 Charging curve of a 100 μF capacitor charged by iTENG driven by cardiac motion and synchronous femoral arterial pressure (FAP) and heart rate (HR) of the Yorkshire porcine.

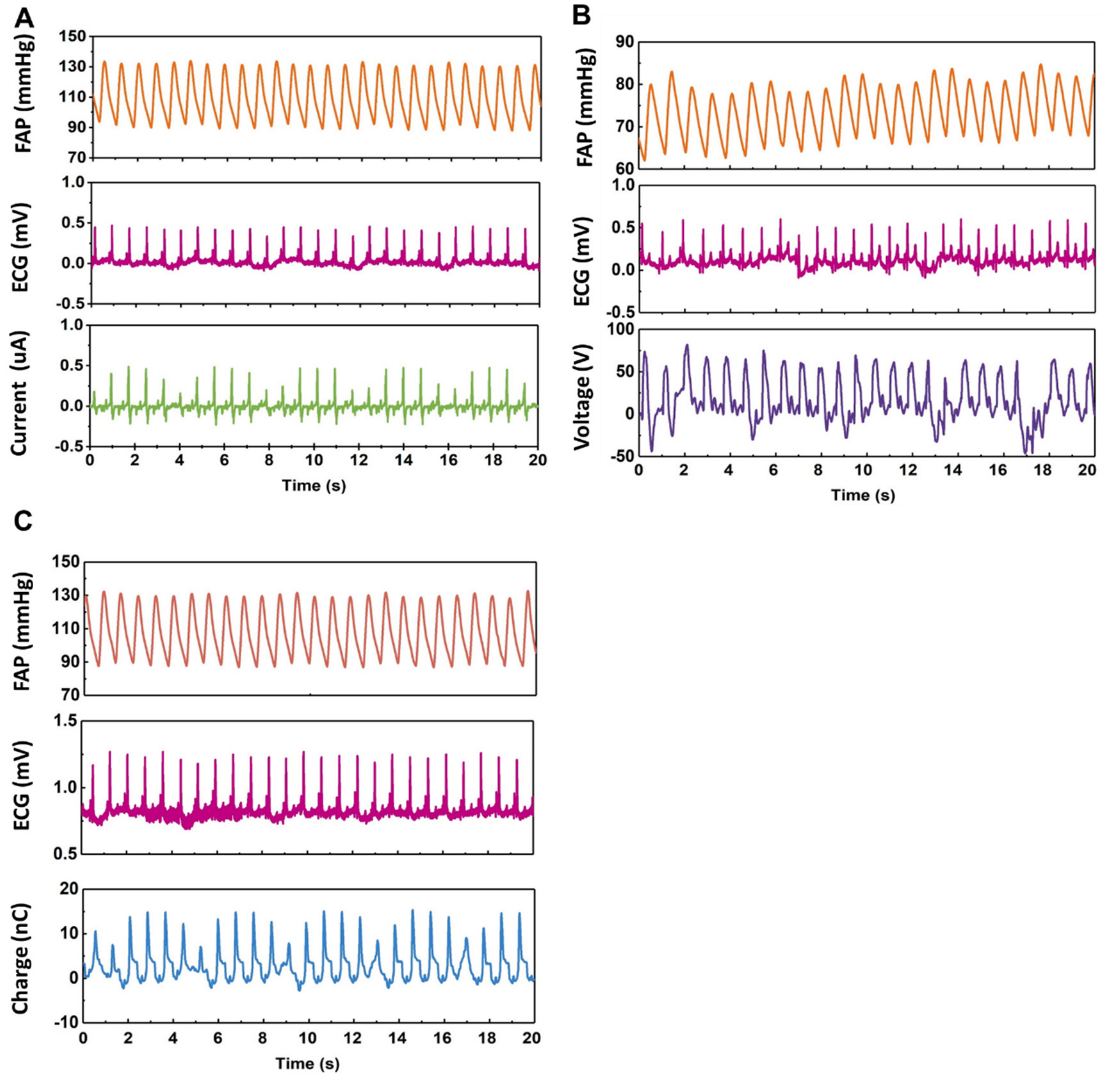

Supplementary Figure 5 *In-vivo* electrical output of iTENG. (a, b, c) *In vivo* open-circuit voltage (a), short-circuit current (b) and transferred charge (c) of the iTENG and simultaneously recorded ECG and FAP of the Yorkshire porcine.

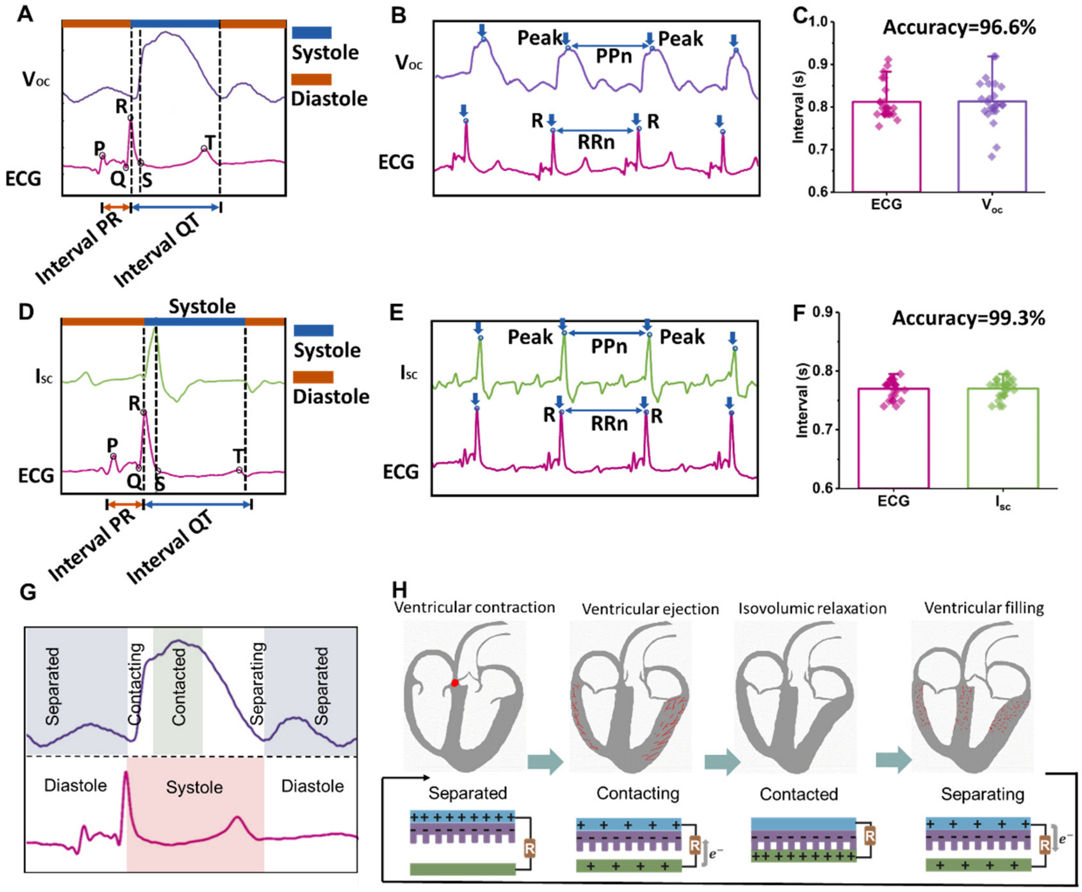

Supplementary Figure 6 Relationship between electrical output and cardiac motion. (a) *In vivo* open-circuit voltage and simultaneously recorded ECG signals. (b, c) Accuracy of open-circuit voltage of the iTENG with ECGs. (d) In vivo output short-circuit current and simultaneously recorded ECG signals. (e, f) Accuracy of short-circuit current of the iTENG with ECGs. (g, h) Relationship between iTENG and cardiac motion. All data in (c, f) are presented as mean ± s.d.

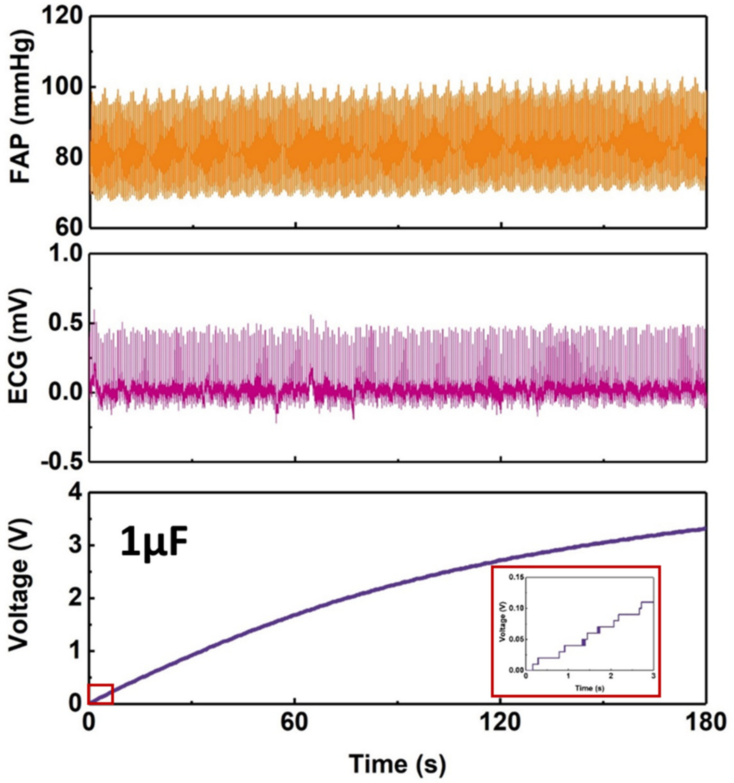

Supplementary Figure 7 Charging curve of a 1 μF capacitor charged by iTENG driven by cardiac motion and synchronous femoral arterial pressure (FAP) and heart rate (HR) of the Yorkshire porcine.

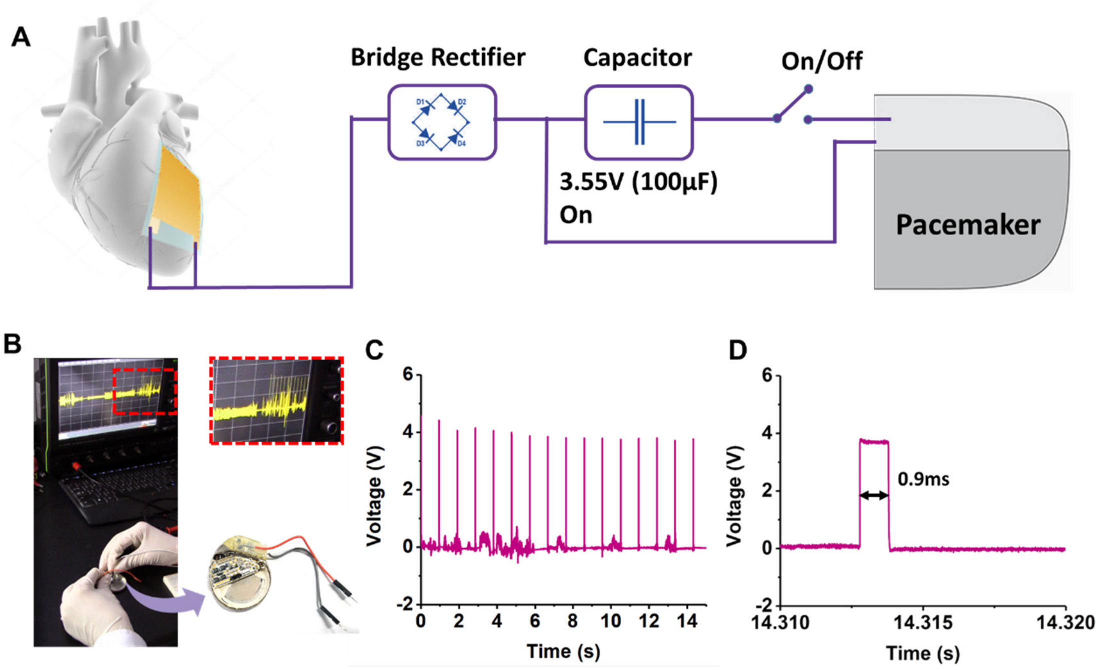

Supplementary Figure 8 Commercial pacemaker drived by iTENG. (a) Energy harvested by iTENG from cardiac motion used to driving commercial pacemaker. (b) Measurement process of pacing pulse signal from commercial pacemaker (c) Pacing pulse signal from commercial pacemaker which powered by capacitance (100 μF, 3.55 V).

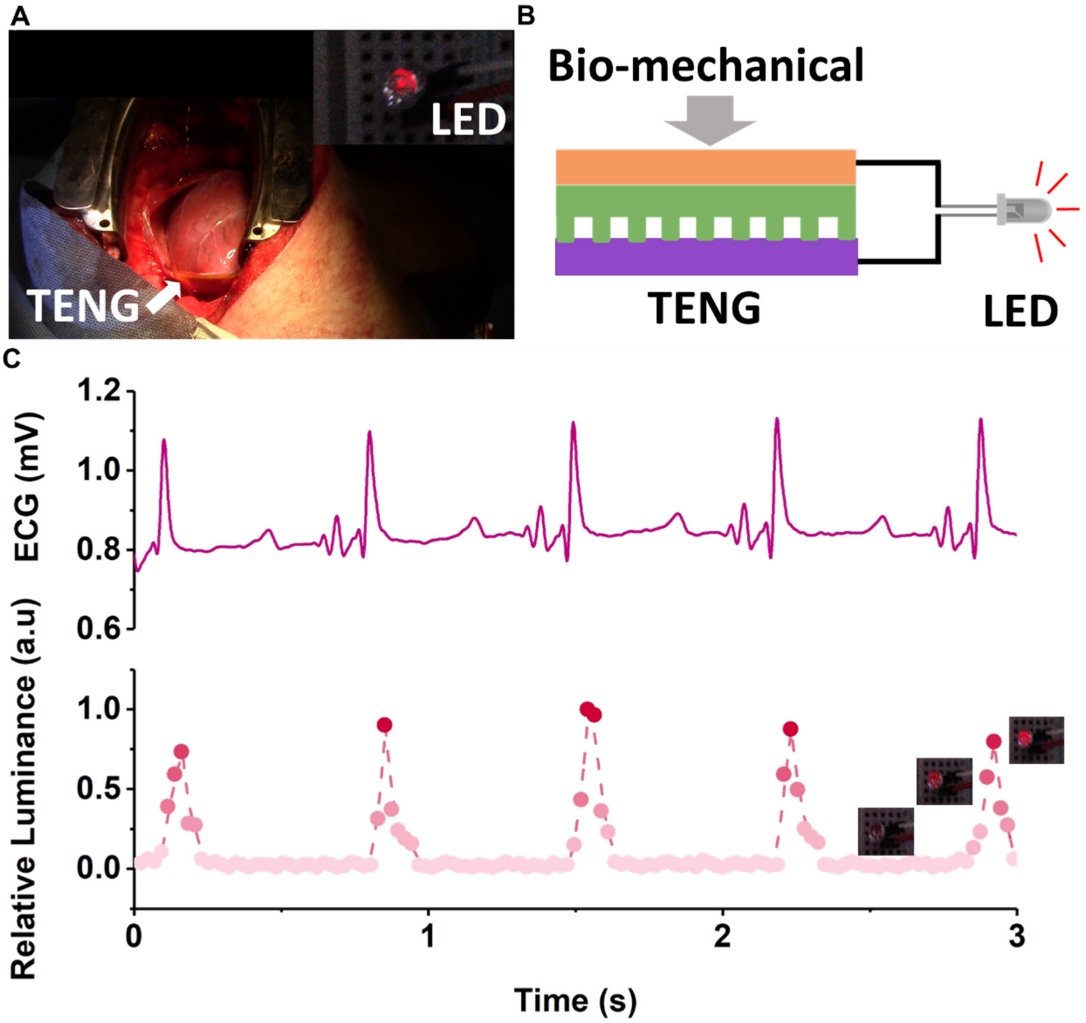

Supplementary Figure 9 LED drived by iTENG. (a) iTENG implanted between the heart and pericardium and blinking of the LED. (b) Illustration of self-powered lighting system based on iTENG. (c) LED relative luminance and synchronized ECG.

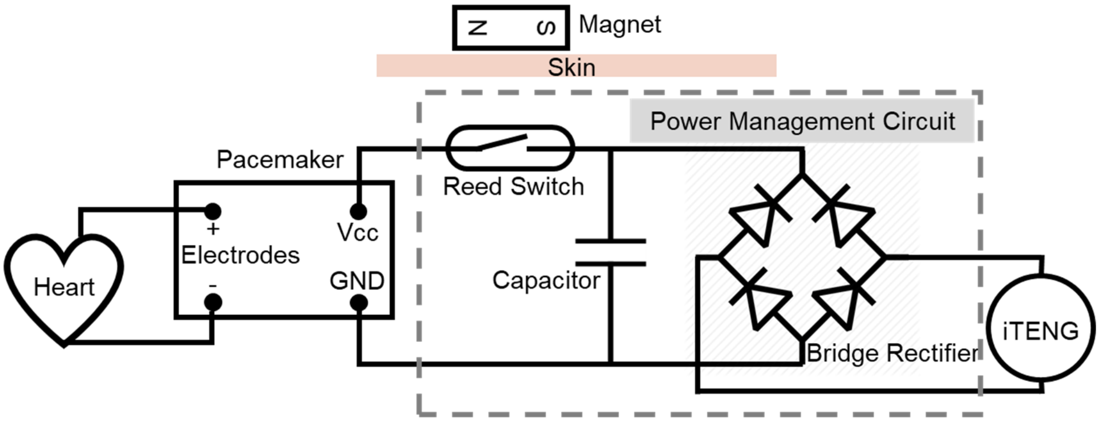

Supplementary Figure 10 Circuit of the symbiotic cardiac pacemaker system.

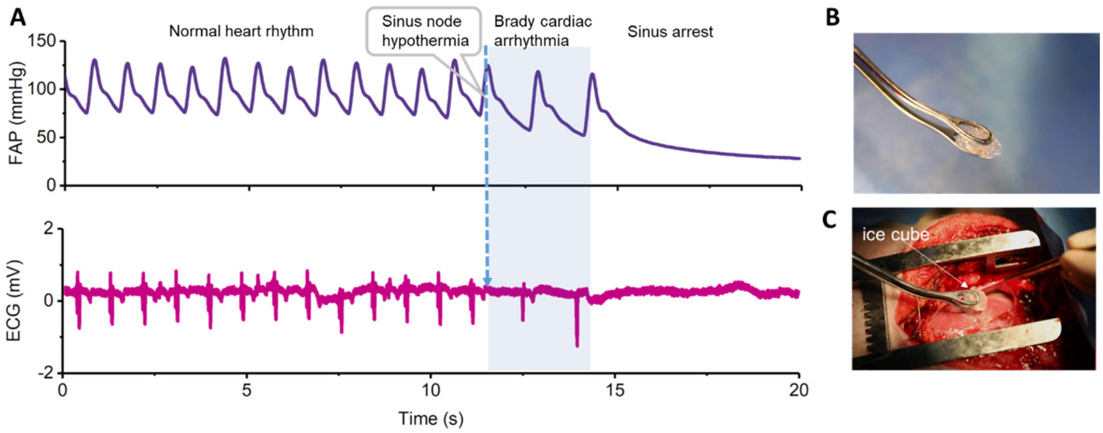

Supplementary Figure 11 Arrhythmia induced by sinus node hypothermia deteriorated to sinus arrest (a) Femoral Artery Pressure (FAP) and ECG of animal model during sinus node hypothermia. (b, c) Sinus node hypothermia induced by ice cube.

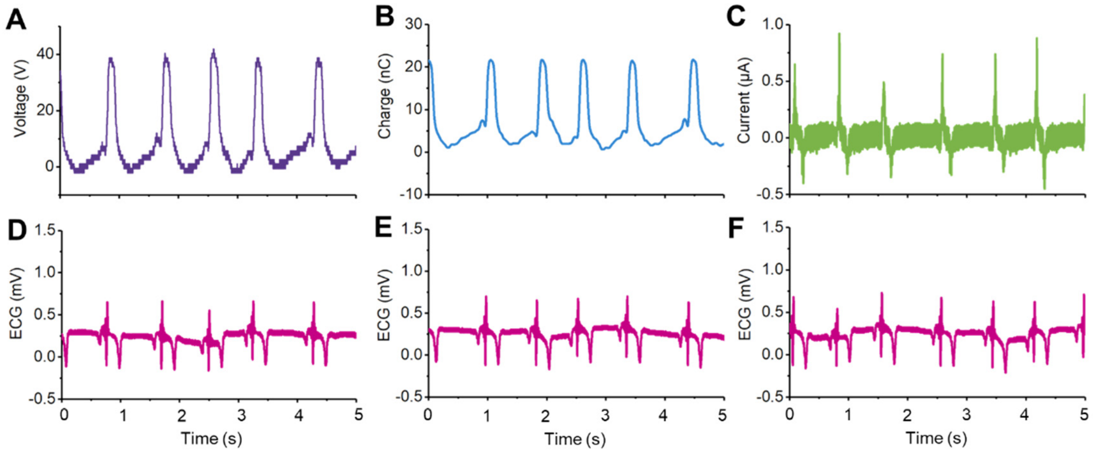

Supplementary Figure 12 *In-vivo* electrical output of iTENG (Correcting ing arrhythmia experiments). (a, b, c) *In vivo* open-circuit voltage (a), transferred charge (b) and short-circuit current (c) of the iTENG and simultaneously recorded ECG of the Yorkshire porcine.

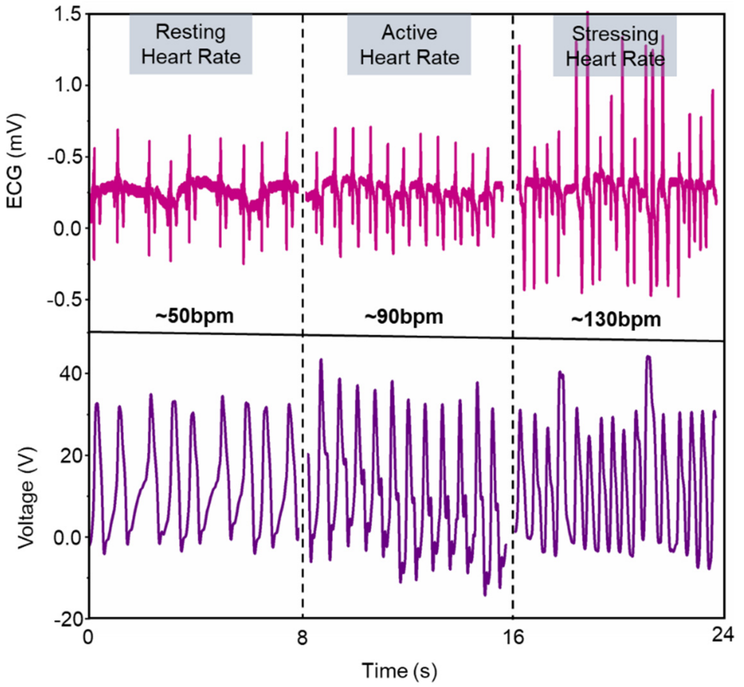

Supplementary Figure 13 Output voltage under different heart states. The consistency between R waves and voltage peaks remained well under resting (~50 bpm), active (~90 bpm), and stressing (~130 bpm) states.

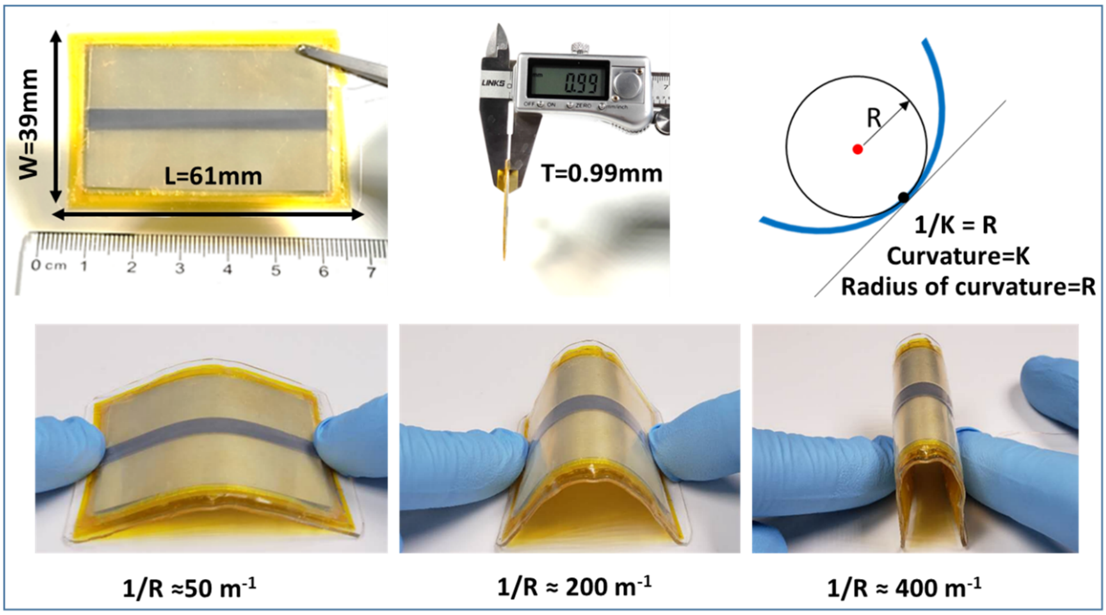

Supplementary Figure 14 Photographs of iTENG. Photographs of iTENG with dimension information.

Supplementary Tables

	Driving mode	Biological regulation application	Model of regulation experiment	Therapeutically effect evaluation	Stimulus signal controllable
**Advanced materials (2014)[1]**	*In Vivo* (drive by breath motion)	Cardiac Pacing	Small animal (rat)	--	Control by pulse generator
**Advanced materials (2014)[2]**	*In Vitro* (drive by human hand)	Cardiac stimulate	Small animal (rat)	--	--
**PNAS (2014)[3]**	*In vivo* (drive by stomach motion	--	Human-scale animal (pig)	--	--
**Energy Environ. Sci (2015)[4]**	*In Vitro* (drive by human hand)	Brian stimulate	Small animal (rat)	--	--
**Nano Energy (2017)[5]**	*In Vitro* (drive by linear motor)	nerves stimulate	Small animal (rat)	--	--
**Nano Energy (2018)[6]**	*In Vitro* (drive by linear motor)	tissue engineering	Cell (fibroblast cell)	--	--
**Nature Communications (2018)[7]**	*In vivo* (drive by heart, lung, and diaphragm motion	nerves stimulate	Small animal (rat)	Obesity treatment	--
**This Work**	*In vivo* (drive by cardiac motion	Cardiac Pacing	Human-scale animal (pig)	Arrhythmia treatment	Control by pacemaker unit

Supplementary Table 1 Comparison of the implantable self-powered device in this work and previous works in recent years for biological regulation.

Output performance	Keel/3D sponge spacer	Keel	3D sponge spacer	PDMS spacer
V_{OC} (V)	97.5	61.5	71.3	50.8
I_{SC} (μA)	10.1	9.68	5.93	5.02
Q_{SC} (nC)	49.1	32.2	41.8	25.9

Supplementary Table 2 Electrical output statistical result of iTENG with different supporting structures.

References (year)	Advanced materials (2014) [1]	Science advances (2016) [8]	Nano letter (2016)[9]	ACS Nano (2016)[10]	Advanced materials (2018) [11]	Nature Communications (2018) [7]	This Work
**Supporting structure**	Spacer	Spacer	Spacer	Spacer/ Kneel	Spacer	Arch	Sponge Spacer/ Kneel
**Surface of triboelectric layer**	Micro pyramid	Nanopillar	Nanopillar	Nanopillar	Nanopillar	Nanopillar	Nanopillar/ polarized
**Maximum output voltage *in vivo***	4 V	4 V	10 V	14 V	4.5 V	0.12 V	65.2 V
**Driving force**	Breath motion	Hand tapping	Cardiac motion	Cardiac motion	Hand tapping	Stomach Motion	Cardiac motion
**Durability reported**	--	--	--	--	--	--	100 million cycles
**Animal model**	Rat	Rat	Pig	Pig	Rat	Rat	Pig

Supplementary Table 3 Comparison of the implantable energy harvesters based on triboelectric effects.

Types	Triboelectric devices	Piezoelectric devices	Electromagnetic devices	Biofuel cells	Endocochlear Potential cells
Materials	Two materials with different tribopolarities	Piezoelectric materials	Metal coils and magnets	Enzyme/metal electrodes	Glass microelectrodes
Output voltage (V)*	High (10 - 65.2)	High (3 - 17.8)	Low (0.1 - 0.9)	Low (0.2 - 0.6)	Low (0.07 - 0.1)
Output power (μW)	0.64	0.72 - 1.08	0.78 - 1.7	7.45 - 10	1.1 - 6.3 nW
Flexible or Rigid	Flexible	Flexible	Rigid	Rigid	Rigid
Weight (g)**	Light (1.9)	Light	Weight (7.2 - 16.7)	Weight (6)	Light
Durability reported	100 million cycles	20 million cycles	Long (several years)	Several months	5 h
Cost	Low	High	Low	Low	High
Ref.	[9,12] and this manuscript	[13,14]	[15,16]	[17-19]	[20]

Supplementary Table 4 Comparison main methods of implantable energy harvesters. *Low < 3 V < High; 3 V is according to voltage of pacemaker battery. **Light < 2 g < Weight; 2 g is according to the weight of miniaturized commercial leadless pacemaker.

Length (mm)	Width (mm)	Thickness (mm)	basal area (cm^2)	Volume (cm^3)	Mass (g)
61	39	0.99	23.8	2.36	1.9

Supplementary Table 5 Dimension information of iTENG

Supplementary Notes

Supplementary Note 1

Principle and process of contact electrification

Contact electrification is main caused by the transfer of surface electrons[21] (Fig.1 g). When an external force was applied, two triboelectric layers contacted to each other, resulting in the generation of free electrons between two triboelectric layer surfaces moving to a single potential well. With distance of two triboelectric layers increasing, a smaller barrier was formed and electrons kept moving between the surfaces. Along with the distance continued to increase, the probability of electrons jumping to the opposite surface became quite small, thus the electrons resided on one surface. When the distance reached far enough, the barrier was high to trap the electrons on different triboelectric layer surfaces (Fig. 1h, i). Here, the potential energy of transferring electrons between the two surfaces contained two short-range interactions and remote coulomb interaction. The energy difference ΔE was the electrostatic contribution, which also included the local interaction between electrons and the near surface[22].

Supplementary Note 2

Electrical output of iTENG with different supporting structures.

The V-Q-x relationship of contact-mode iTENG can be derived based on electrodynamics[23]. Since the area size (S) of the metals is several orders of magnitude larger than their separation distance $(d + x)$ in the experimental case, it is reasonable to assume that the two electrodes are infinitely large. Under this assumption, the charges on the metal electrodes will uniformly distribute on the inner surfaces of the two metals. Inside the dielectrics and the air gap, the electric field only has the component in the direction perpendicular to the surface, with the positive value pointing to Metal[23].

$$V_{OC} = \frac{\sigma x(t)}{\varepsilon_0} \quad (1)$$

$$Q_{SC} = \frac{S\sigma(t)}{d + x(t)} \quad (2)$$

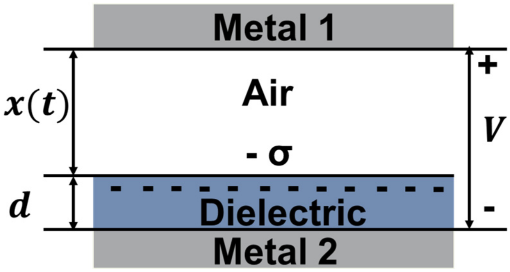

$$I_{SC} = \frac{dQ_{SC}}{dt} = \frac{S\sigma dv(t)}{(d+x(t))^2} \qquad (3)$$

The keel structure significantly strengthened the mechanical property of the overall structure and effectively guaranteed the contact and separation process of the iTENG[10]. Thus, the iTENG with keel structure will possess higher V(t) and x to improve I_{SC} and V_{OC}.

3D sponge spacer supporting structures could effectively increase the contact area Thus, the iTENG with 3D sponge spacer possessed higher S and σ to improve output Voltage. However, the V(t) may decrease with application of 3D sponge spacer which lead to a lower I_{SC}.

Supplementary Note 3

Relationship between electrical output of iTENG and cardiac cycle

We studied the relationship between electrical output of iTENG and cardiac cycle based on synchronized ECG. Accuracy (A) was used to evaluate iTENG's ability to identify a cardiac cycle. The accuracy (A) was defined as the degree of proximity of interval of Peak (PPn) of iTENG signals to interval of R wave (RRn).

$$A = \frac{PP_n - RR_n}{RR_n} \qquad (4)$$

$$\overline{A} = \frac{\sum_1^n A}{n} \qquad (5)$$

As a result, high accuracy of 99.3% (I_{SC}) and 96.6% (V_{OC}) were achieved (Supplementary Fig. 5c, f). It implied that iTENG can not only be used as an energy harvesting device but also as self-powered active sensor for identifying cardiovascular events. The proposal was expected to further which is expected to get rid of relying on amplifying circuits in the traditional ECG mode of cardiac pacemaker.

Supplementary Note 4

Calculation of the energy harvested during each cardiac motion cycle

The generated energy during per cycle was an important parameter that iTENG as

energy harvesting device apply to implantable electronics. The iTENG was driven by heart beat to do continuous periodic mechanical motion. The electrical output was also periodically time-dependent. The average output power $\overline{P}$ was related to the load resistance. Given a certain period of time T, the maximum energy output per cycle of iTENG can be derived by the following equation [24] .

$$E = \overline{P}T = \int_0^T VIdt = \int_{t=0}^{t=T} VdQ = \oint VdQ \tag{6}$$

$$E_{max} = \frac{1}{2} Q_{SC,\,max}(V_{OC,\,max} - V_{OC,\,min}) \tag{7}$$

$$E_{max} = \frac{1}{2} Q_{SC,\,max} \Delta V \tag{8}$$

Here, E_{max} is the maximal output energy per cycle. Through the statistical analysis of the measurement results *in vivo*, the maximal short-circuit transferred charge $Q_{SC,max}$ = 13.6 nC, the voltage difference ΔV = 72.9 V (Fig. 4f, g). So it could be inferred that the $\mathbf{E_{max}}$ **= 0.495 μJ** (*In vivo* energy harvest experiment). The maximal short-circuit transferred charge $Q_{SC,max}$ = 21 nC, the voltage difference ΔV = 41 V (Supplementary Figure 12), thus the $\mathbf{E_{max}}$ **= 0.430 μJ** (Correcting Arrhythmia Experiments)

Supplementary Note 5

Pacing threshold energy

Pacing threshold energy is important to evaluate the feasibility of a self-powered cardiac pacing system from the energy perspective. Here, pacing threshold energy can be derived from the follow equation.

$$E_t = \int_0^T V_t \times Id_t \tag{9}$$

Since the pacing pulse is a square wave, the equation can be simplified as follow.

$$E_t = V_t \times I \times T = \frac{V_t^2 \times T}{R} \tag{10}$$

Here E_t is the pacing threshold energy. V_t represents the pacing threshold voltage. R represents the pacing resistance. T stands for stimulus pulse durations.

As recent research visit for the 60 patients shows that the mean pacing capture

threshold measured at 0.24 ms. the mean electrical values for R-wave sensing amplitude, pacing impedance, and pacing capture threshold at 0.24 ms were, respectively: 11.7 ± 4.5 mV, 719 ± 226 Ω, 0.57 ± 0.31 V at implant[25]. E_t is 0.197 ± 0.18 μJ. The maximum pacing threshold energy (E_t) is about 0.377 μJ.

On the other hand, the pacing threshold voltage of pigs is 0.7 V at 0.5 ms , the pacing impedance = 953 Ω. The pacing threshold energy is about 0.262 μJ[26].

Supplementary Note 6

Output voltage and power of iTENG under different heart states

we record the output voltage of iTENG under resting (~50 bpm), active (~90 bpm), and stressing (~130 bpm) states (Adult Yorkshire porcine, male, 35 kg, in correcting arrhythmia experiments). The peak voltages of iTENG were about 32 V, 38 V, and 31 V under resting (~50 bpm), active (~90 bpm), and stressing (~130 bpm) states, respectively. The output voltage amplitudes have little changes, and the output power would increase with the heart rate if the strength of heart beat kept constant.

In this specific application, we have obtained the energy harvested in each cycle E_H = 0.430 μJ (ΔV_{OC} = 41 V, Q = 21 nC, ~82 bpm), in correcting arrhythmia on large animal model experiment (Supplementary Note 4 Calculation of the energy harvested during each cardiac motion cycle).

The output power can be derived from the following equation:

$$E_H = \frac{1}{2} Q_{SC,max} \Delta V \tag{11}$$

$$Q_{iTENG} = C_{iTENG} V_{iTENG} \tag{12}$$

$$E_H = \frac{1}{2} C_{iTENG} V_{OC,max} \Delta V \tag{13}$$

$$P = E_H \times HR/60 \tag{14}$$

Here, the C_{iTENG} is the capacitance of the iTENG. Thus the output power of iTENG were as follows:

P = 0.218 μW = 50 bpm × 0.262 μJ, under resting state (~50 bpm);

P = 0.555 μW = 90 bpm × 0.370 μJ, under active state (~90 bpm);

P = 0.533 μW = 130 bpm × 0.246 μJ, under stressing state (~130 bpm).

The maximum ouput power of iTENG was 0.553 μW under active state of heart.

Heart state (HR)	Resting (~50 bpm)	Active (~90 bpm)	Stressing (~130 bpm)
Output voltages	31 V	38 V	32 V
Output Power	0.218 μW	0.555 μW	0.533 μW

(The output voltage and power have been evaluated under resting (~50 bpm), active (~90 bpm), and stressing (~130 bpm) states)

Supplementary Note 7

Calculation of energy efficiency for iTENG

The energy efficiency of iTENG in pacing experiment can be estimated according to the following formula:

$$\text{Energy efficiency } (\eta) = \frac{\text{Stored Energy}}{\text{Harvested Energy}} \quad (15)$$

Therefore, all energy harvested from the heart is:

$$E_H = E_{H,\text{per cycle}} \times T \times HR = 0.495\ \mu J \times 200\ \text{min} \times 77\ \text{bpm} = 7623\ \mu J \quad (16)$$

The energy stored in the energy management unit is:

$$E_{\text{stroed}} = \frac{1}{2} \times C \times V^2 = \frac{1}{2} \times 100\ \mu F \times (3.55\ V)^2 = 630.1\ \mu J \quad (17)$$

$$\eta = \frac{E_{\text{stroed}}}{E_{\text{harvested}}} = \frac{630.1\ \mu J}{7623\ \mu J} = 8.3\ \% \quad (18)$$

Thus, the stored energy is 630.1 μJ and the energy efficiency is 8.3 %.

Supplementary Note 8

Commercial pacemaker drived by iTENG

The iTENG was connected to a 100 μF capacitor through a rectifier. Within 190 min, the voltage of capacitor be charged from 0 to 3.55 V. The electric energy was driving the commercial pacemaker (Adapta, ADDR03, Medtronic) produce the stimulus pulses signals. The voltage was detected by an electrometer (Keithley 6517B) and recorded by oscilloscope (Teledyne LeCroy HD 4096).

Supplementary Note 9

LED drived by iTENG

The LEDs were directly connected with iTENG that was implanted between the heart and pericardium. LED light signal was captured by the camera. The relative luminous intensity of the LED light-emitting region was analyzed using image analysis software (Image-Pro Plus 6.0).

Supplementary References

1 Zheng, Q. *et al.* In Vivo Powering of Pacemaker by Breathing-Driven Implanted Triboelectric Nanogenerator. *Adv Mater* **26**, 5851-5856 (2014).

2 Hwang, G.-T. *et al.* Self-Powered Cardiac Pacemaker Enabled by Flexible Single Crystalline PMN-PT Piezoelectric Energy Harvester. *Adv Mater* **26**, 4880 (2014).

3 Dagdeviren, C. *et al.* Conformal piezoelectric energy harvesting and storage from motions of the heart, lung, and diaphragm. *P Natl Acad Sci USA* **111**, 1927-1932, (2014).

4 Hwang, G. T. *et al.* Self-powered deep brain stimulation via a flexible PIMNT energy harvester. *Energy & Environmental Science* **8**, 2677-2684 (2015).

5 Lee, S. *et al.* Development of battery-free neural interface and modulated control of tibialis anterior muscle via common peroneal nerve based on triboelectric nanogenerators (TENGs). *Nano Energy* **33**, 1-11(2017).

6 Wang, A. C. *et al.* Piezoelectric nanofibrous scaffolds as in vivo energy harvesters for modifying fibroblast alignment and proliferation in wound healing. *Nano Energy* **43**, 63-71 (2018).

7 Yao, G. *et al.* Effective weight control via an implanted self-powered vagus nerve stimulation device. *Nat Commun* **9**, 5349, (2018).

8 Zheng, Q. *et al.* Biodegradable triboelectric nanogenerator as a life-time designed implantable power source. *Science Advances* **2**, e1501478 (2016).

9 Ma, Y. *et al.* Self-powered, one-stop, and multifunctional implantable triboelectric active sensor for real-time biomedical monitoring. *Nano Lett* **16**, 6042-6051 (2016).

10 Zheng, Q. *et al.* In Vivo Self-Powered Wireless Cardiac Monitoring Via Implantable Triboelectric Nanogenerator. *ACS nano* (2016).

11 Zhang, L. *et al.* Low Interface Energies Tune the Electrochemical Reversibility of Tin Oxide Composite Nanoframes as Lithium-Ion Battery Anodes. *Acs Applied Materials & Interfaces* **10**, 36892-36901 (2018).

12 Zheng, Q. *et al.* In vivo self-powered wireless cardiac monitoring via implantable triboelectric nanogenerator. *ACS nano* **10**, 6510-6518 (2016).

13 Dagdeviren, C. *et al.* Conformal piezoelectric energy harvesting and storage from motions of the heart, lung, and diaphragm. *Proceedings of the National Academy of Sciences* **111**, 1927-1932 (2014).

14 Lu, B. *et al.* Ultra-flexible piezoelectric devices integrated with heart to harvest the biomechanical energy. *Scientific reports* **5**, 16065 (2015).

15 Zurbuchen, A. *et al.* Endocardial Energy Harvesting by Electromagnetic Induction. *IEEE transactions on biomedical engineering* **65**, 424-430 (2018).

16 Zurbuchen, A. *et al.* Towards batteryless cardiac implantable electronic devices—the Swiss way. *IEEE transactions on biomedical circuits and systems* **11**, 78-86 (2017).
17 Halámková, L. *et al.* Implanted biofuel cell operating in a living snail. *Journal of the American Chemical Society* **134**, 5040-5043 (2012).
18 El Ichi, S. *et al.* Bioelectrodes modified with chitosan for long-term energy supply from the body. *Energ Environ Sci* **8**, 1017-1026 (2015).
19 Shoji, K. *et al.* Biofuel cell backpacked insect and its application to wireless sensing. *Biosensors and Bioelectronics* **78**, 390-395 (2016).
20 Mercier, P. P., Lysaght, A. C., Bandyopadhyay, S., Chandrakasan, A. P. & Stankovic, K. M. Energy extraction from the biologic battery in the inner ear. *Nature biotechnology* **30**, 1240 (2012).
21 Xu, C. *et al.* On the Electron-Transfer Mechanism in the Contact-Electrification Effect *Adv Mater* ***27*** 0489 (2018).
22 McCarty, L. S. & Whitesides, G. M. Electrostatic charging due to separation of ions at interfaces: contact electrification of ionic electrets. *Angewandte Chemie International Edition* **47**, 2188-2207 (2008).
23 Niu, S. *et al.* Theoretical study of contact-mode triboelectric nanogenerators as an effective power source†. *Energy and Environmental Science* **6**, 3576-3583 (2013).
24 Zi, Y. *et al.* Standards and figure-of-merits for quantifying the performance of triboelectric nanogenerators. *Nature communications* **6** (2015).
25 Ritter, P. *et al.* Early performance of a miniaturized leadless cardiac pacemaker: the Micra Transcatheter Pacing Study. *European heart journal* **36**, 2510-2519 (2015).
26 Furrer, M. *et al.* VATS-guided epicardial pacemaker implantation - Hand-sutured fixation of atrioventricular leads in an experimental setting. *Surg Endosc-Ultras* **11**, 1167-1170 (1997).
