# Supplementary material for: Symbiotic cardiac pacemaker
Source: Nat Commun. 2019 Apr 23;10:1821. doi: 10.1038/s41467-019-09851-1 (PMC6478903; doi:10.1038/s41467-019-09851-1)
Supplement: Supplementary file 7 — Description of Additional Supplementary Files [file 41467_2019_9851_MOESM7_ESM.pdf]

Description of Additional Supplementary Files

File Name: Supplementary Movie 1

Description: Capacitor (100μF) charged by iTENG.

File Name: Supplementary Movie 2

Description: A LED powered by iTENG in vivo.

File Name: Supplementary Movie 3

Description: Cardiac pacing on large animal model by SPM.
